# Successful Treatment of Evans Syndrome Onset With Zanubrutinib in Combination With Romiplostim During Venetoclax Treatment in a Patient With Chronic Lymphocytic Leukemia: A Case Report

**DOI:** 10.1002/ccr3.72697

**Published:** 2026-05-18

**Authors:** Vanessa Innao, Alessia Paola Maria Barbagallo, Oriana Bianco, Valerio Leotta, Ugo Consoli

**Affiliations:** ^1^ U.O.C di Ematologia Azienda Ospedaliera di Rilievo Nazionale e di Alta Specializzazione, ARNAS Garibaldi di Catania Catania Italy

**Keywords:** chronic lymphocytic leukemia, Evans syndrome, immune thrombocytopenia, romiplostim, thrombopoietin receptor agonist, zanubrutinib

## Abstract

Chronic lymphocytic leukemia (CLL) can be complicated by autoimmune cytopenias, including autoimmune hemolytic anemia and immune thrombocytopenia (ITP), as observed in Evans syndrome. We report the case of a 75‐year‐old man with CLL who developed steroid‐ and rituximab‐refractory Evans syndrome during the ramp‐up phase of venetoclax treatment. The patient presented worsening of preexisting subclinical hemolysis that had been present before starting venetoclax, along with severe ITP (platelets 0 × 10^9^/L), unresponsive to standard therapies including steroids, immunoglobulins, and rituximab. Rescue treatment with the combination of romiplostim, a thrombopoietin receptor agonist, and zanubrutinib, a second‐generation Bruton's tyrosine kinase inhibitor, led to complete and sustained platelet recovery within 4 weeks. This case highlights the potential of combining zanubrutinib with a thrombopoietin receptor agonist in managing severe ITP secondary to CLL, particularly when conventional therapies fail.

## Key Clinical Message

Combination therapy with zanubrutinib and romiplostim can achieve rapid and sustained hematologic response in severe immune thrombocytopenia secondary to chronic lymphocytic leukemia, particularly when standard immunosuppressive treatments fail. BTK inhibition may play a central role in controlling immune dysregulation, driving Evans syndrome in patients with chronic lymphocytic leukemia.

## 1 Introduction

Chronic lymphocytic leukemia (CLL) is the most common leukemia in western countries [1], with an age‐related incidence and high clinical variability. Evans syndrome (ES) is an immune disorder characterized by the simultaneous or subsequent development of immune thrombocytopenia (ITP) and autoimmune hemolytic anemia [2]. This condition can be associated with lymphoproliferative disorders and sometimes can have a fatal outcome [2]. There are no randomized trials or guidelines, but only consensus‐based expert recommendations for the management of these patients [3]. Standard therapies for treating CLL include Bruton's kinase inhibitors (BTKis) ibrutinib, acalabrutinib, and zanubrutinib, and BCL2 inhibitor venetoclax, both in combination or with anti‐CD20 rituximab or obinutuzumab [1]. When CLL presentation or progression is associated with an immune cytopenia (anemia and/or thrombocytopenia), the emergence of a life‐threatening condition led to treating patients with high‐dose steroids alone or in combination with immunoglobulins, rituximab, or immunosuppressive drugs and starting anti‐CLL therapy in refractory cases. However, although autoimmune cytopenias are well‐recognized complications of CLL, published data on severe immune thrombocytopenia/Evans syndrome arising during venetoclax treatment and subsequently managed with Zanubrutinib in combination with romiplostim remain limited.

## 2 Case History

We describe the case of a 75‐year‐old man, elsewhere diagnosed in 2019 with asymptomatic classical CLL, who developed in February 2021 a hemolytic anemia with positivity of Coombs tests (warm autoimmune hemolytic anemia), refractory to steroids and high doses of immunoglobulins. The patient started the first CLL therapy line with bendamustine plus rituximab for six cycles, achieving a partial response. In July 2024, the patient showed a rapid progression of lymphocytosis, with a doubling time of less than 2 months, associated with mild thrombocytopenia (PLT 86 × 10^9^/L), worsening splenomegaly (23 cm), and constitutional symptoms (night sweats and weight loss), consistent with stage IV Rai and C Binet. The biological assessment showed the absence of FISH alterations, IGHV status unmutated, and *TP53* wild‐type, so the CLL‐IPI (Chronic Lymphocytic Leukemia—International Prognostic Index) score was 6 (high risk). Therefore, he started a second‐line CLL therapy with venetoclax and rituximab, according to the Murano schedule. The tumor lysis syndrome risk score was high, but despite good tolerance in the absence of tumor lysis syndrome signs, during the second week of the ramp‐up phase, the patient experienced the onset of spontaneous ecchymoses and gingival bleeding. Laboratory tests showed PLT 0 × 10^9^/L, a positive nasopharyngeal swab for COVID‐19, as well as positive direct and indirect Coombs tests, supporting autoimmune hemolysis. Hemoglobin was 10.7 g/dL, and the white blood cell count was nearly within normal range.

## 3 Differential Diagnosis, Investigations, and Treatment

Peripheral blood smear did not show schistocytes, thus arguing against thrombotic microangiopathy, and bone marrow evaluation showed megakaryopoiesis and no evidence of marrow aplasia/failure, explaining the abrupt severe thrombocytopenia. Thus, the medical team decided to discontinue venetoclax and initiate steroid and immunoglobulin therapy promptly, but there was no response. Therefore, after antiviral treatment and negative conversion of the swab, the patient underwent rituximab therapy at a dose of 375 mg/mq weekly for four administrations. However, platelet levels showed no improvement, and the patient was therefore referred to our center, a hub specializing in the treatment of immune thrombocytopenia.

After evaluating the case and considering the autoimmune origin of the thrombocytopenia in a patient with positive Coombs tests, we decided to initiate rescue therapy by combining the subcutaneous thrombopoietin receptor agonist (TPO‐RA) romiplostim with a BTKi, opting for zanubrutinib. This choice was supported by the results of the ALPINE study, which reported that zanubrutinib was superior to ibrutinib in efficacy and safety, particularly with respect to cardiovascular adverse events [4].

## 4 Conclusion and Results

**FIGURE 1:**
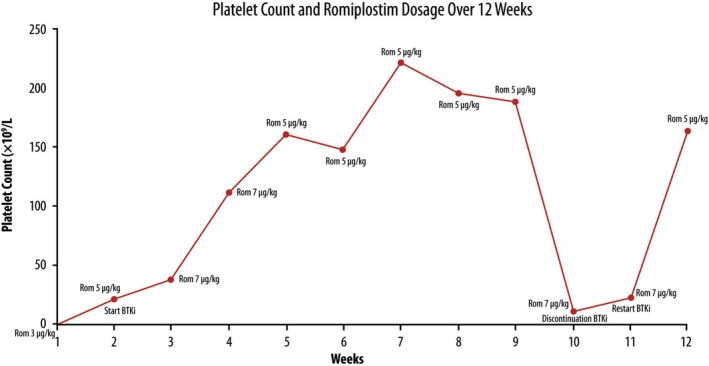
Platelet count over 12 weeks in a patient with secondary ITP treated with romiplostim and zanubrutinib. Dosage adjustments for romiplostim and key treatment events (BTKi initiation, discontinuation and reintroduction) are annotated. Platelet normalization was achieved by week 4, with sustained response following combination therapy.

After the first dose of romiplostim at dosage of 3 μg/kg/weekly the platelets were 21 × 10^9^/L, so TPO‐RA was gradually increased by 2 μg/kg/weekly, so starting zanubrutinib from the second week at a dose of 160 mg twice daily, achieving platelet normalization (PLT > 100 × 10^9^/L) within 4 weeks (Figure 1). Following this response, the patient continued full‐dose BTKi therapy up to the present day—more than 6 months after the onset of severe thrombocytopenia—and tapered the TPO‐RA to a maintenance dose of 5 μg/kg per week. It is noteworthy that BTKi therapy was interrupted for 10 days due to drug unavailability, during which thrombocytopenia (PLT 11 × 10^9^/L) recurred despite ongoing TPO‐RA treatment, and platelet counts recovered upon reintroduction of zanubrutinib. Since then, the patient has maintained a complete hematologic response (PLT > 100,000/mmc) and has shown a reduction in splenomegaly, with full recovery of hematopoiesis, without any reported adverse events or toxicity to date, including thrombosis and cardiovascular or bleeding complications.

In conclusion, TPO‐RAs represent a highly effective class of drugs for the treatment of primary ITP [5]. However, it is well known that in secondary ITP driven by an underlying lymphoproliferative disorder, targeting only the latter results in more sustained responses. In fact, BTKis alone have proven extremely effective in the treatment of ITP, as demonstrated with rilzabrutinib in the Luna 3 study [6, 7], which highlighted the central role of B‐cell control in sustaining the immune‐mediated process. Our case is therefore emblematic, demonstrating that the combination of these two classes of drugs has proven both safe and effective.

## 5 Discussion

Secondary ITP in the context of CLL is a rare but severe complication. Although severe thrombocytopenia occurred during venetoclax ramp‐up, a direct causal relationship cannot be established from a single case. Several factors may have contributed to the onset of Evans syndrome in our patient, including preexisting autoimmune hemolysis, active CLL‐related immune dysregulation, and concomitant COVID‐19 infection. Therefore, venetoclax should be regarded as a possible temporal trigger in a biologically susceptible context rather than a proven causal factor. TPO‐RAs have demonstrated efficacy in primary ITP, but their role in secondary forms, especially those associated with lymphoproliferative disorders, remains less defined. Recent studies have emphasized the potential of BTKis as immune modulators.

Beyond disease control in CLL, BTK inhibition may have immunomodulatory effects relevant to autoimmune cytopenias, including attenuation of autoreactive B‐cell signaling and modulation of Fc receptor‐dependent macrophage‐mediated platelet clearance. Prior reports with ibrutinib in CLL‐associated autoimmune cytopenias and the activity of rilzabrutinib in ITP indirectly support this therapeutic rationale [6, 7, 8]. In our patient, zanubrutinib was selected because it could simultaneously target the underlying lymphoproliferative disorder and the immune dysregulation, while offering a favorable safety profile. Conversely, venetoclax rechallenge was not considered optimal in the acute phase because of the close temporal association with the event, and additional immunosuppressive strategies were considered less attractive after failure of steroids, immunoglobulins, and rituximab.

The relapse of thrombocytopenia during zanubrutinib discontinuation, despite continued TPO‐RA, suggests a central role for BTK inhibition in modulating the immune mechanism underlying Evans syndrome secondary to CLL, which in this case was defined by the simultaneous presence of autoimmune hemolytic anemia—evidenced by reduced hemoglobin, low haptoglobin, positive direct Coombs test, and laboratory signs of hemolysis—and severe thrombocytopenia not attributable to other causes.

In summary, this case suggests that the combination of zanubrutinib and romiplostim may represent a feasible and safe rescue strategy in selected patients with CLL‐associated Evans syndrome or severe secondary immune thrombocytopenia refractory to standard therapies [9]. Given the coexistence of multiple potential triggers, the observation should be interpreted cautiously, but it supports further investigation of BTK inhibitor‐based approaches in this setting.

## Author Contributions

**Vanessa Innao:** conceptualization, methodology, writing – original draft, writing – review and editing. **Alessia Paola Maria Barbagallo:** writing – original draft, writing – review and editing. **Oriana Bianco:** methodology, writing – review and editing. **Valerio Leotta:** methodology, writing – review and editing. **Ugo Consoli:** conceptualization, writing – review and editing.

## Funding

This work was supported by BeOne Medicines Italy S.r.l.

## Ethics Statement

Ethical approval was not required for this case report in accordance with national regulations and institutional policies.

## Consent

Written informed consent was obtained from the patient for publication of this case report.

## Conflicts of Interest

The authors declare no conflicts of interest.

## Data Availability

All data generated or analyzed in this case report are included in this article and figure. Further inquiries can be directed to the corresponding author.
